# Integrated optical phased array with on-chip amplification enabling programmable beam shaping

**DOI:** 10.1038/s41598-024-60204-5

**Published:** 2024-04-26

**Authors:** Marco Gagino, Alonso Millan-Mejia, Luc Augustin, Kevin Williams, Erwin Bente, Victor Dolores-Calzadilla

**Affiliations:** 1 Eindhoven Hendrik Casimir Institute, Technical University of Eindhoven, 5612 AP Eindhoven, The Netherlands; 2 SMART Photonics, 5656 AE Eindhoven, The Netherlands

**Keywords:** Photonic integration, Indium Phosphide, Optical phased array, Other photonics, Electrical and electronic engineering

## Abstract

We present an integrated optical phased array (OPA) which embeds in-line optical amplifiers and phase modulators to provide beam-forming capability with gain and beam steering in the 1465–1590 nm wavelength range. We demonstrate up to 21.5 dB net on-chip gain and up to 35.5 mW optical output power. The OPA circuit is based on an InP photonic integration platform and features the highest measured on-chip gain and output power level recorded in an active OPA (i.e., with amplification), to the best of our knowledge. Furthermore, the OPA enables the independent control of both amplitude and phase in its arms and through this we demonstrate programmable beam shaping for two cases. First, we carried out a Gaussian apodization of the power distribution profile in the OPA emitter waveguides, leading to 19.8 dB sidelobe suppression in the far-field beam, which is the highest value recorded for active OPAs, and then we demonstrated beam forming of 0th, 1st, and 2nd order 1D Hermite–Gaussian beams in free-space.

## Introduction

Optical phased arrays have attracted increasing interest in literature for their ability to form and steer a beam in free-space relying on a fully solid-state solution with no moving parts. Beam steering devices are crucial components in sensing systems such as LiDAR^1,2^, in optical wireless communications (OWC)^3^, and in biomedical^4,5^ applications. Among the technologies that are currently allowing beam steering, mechanical-based techniques that rely on rotating mirrors or MEMS devices are popular and their use in commercial LiDAR systems have been investigated in recent years^2,6,7^. The optical phased array (OPA) is a solid-state photonic integration technology for beam forming and steering that relies on the mutual interference between N optical antennas and on their relative phase difference to form a beam in free space in any chosen direction. Due the lack of moving parts, OPAs can provide faster beam steering, and they offer the advantage to be more stable against vibrations compared to their mechanical counterparts^8^. Moreover, OPAs based on photonic integrated chips (PICs) have the potential to reduce the size, weight, and power consumption of sensors relying on beam steering.

PIC-based OPAs have been demonstrated in Silicon, Silicon Nitride, and InP photonic integration platforms achieving image resolutions higher than 1000 points^9–11^, sub-$0.1^\circ$ angular resolution^9,10,12–17^, and wide field of view with more than $120^\circ$ beam steering range^12,16,17^. Typical OPA demonstrations have reported diffraction sidelobe suppression ratios (SLSR) close to 10 dB (Table 1). In OPA applications like LiDAR and optical wireless communication, the SLSR of the beam may limit the system performance, thus its maximization is crucial and can be achieved by applying a power distribution such as the Gaussian envelope to the OPA emission profile^18^. Moreover, a wide wavelength span of OPA operation is of interest for dispersion-based 2D beam steering^19–21^ as well as spectral LiDAR applications^22^, and up to 120 nm range of operation has been demonstrated in the C band of the spectrum^15^. Despite the impressive beam forming and steering performances reported in literature, all OPAs suffer from optical losses that limit the output power levels, which are typically caused by propagation and insertion losses of the photonic devices, as well as the fiber-to-chip coupling losses. OPAs with high output optical powers are desirable for applications that require steering and sensing at large distances to compensate for atmospheric absorption, as well as scattering from diffusive targets, and up to 400 mW output power was achieved in a SiN integrated photonic platform with Watt-level optical input power^9,10^. As Table 1 shows though, the OPA transmission losses are 8.1 dB at best, and can be as high as 43 dB^23^, thus limiting the output power level of the free-space beam and imposing stringent requirements on the design of high-power lasers.

To overcome the on-chip optical losses, OPA circuits with embedded SOAs have been demonstrated^24–26^, however their performance in terms of optical amplification was not presented or, as in the case developed by Guo et al.^27^, the SOA gain characterization was performed on a separate test structure, demonstrating up to 35 dB/mm maximum gain and a maximum output power generated by a single SOA of 20 mW. A similar PIC was reported in a high-power laser relying on coherent beam combining^28^. The presence of in-line amplifiers in the OPA arms is not only beneficial in terms of optical output power, but the concept can be exploited to allow the independent control of the amplitude and phase in each arm, which enables the generation of different beam shapes with a single device. Milanizadeh et al.^29^ demonstrated this with a programmable PIC that routes the optical power to selected free-space antennas. Among the beam shapes that can potentially be generated in this way, the Hermite–Gauss (HG) beams are of interested in OPA applications for the emission of multiple high-order beams that can be simultaneously steered. This capability does not require additional circuit complexity of the beam steering device, as opposed to other state of the art solutions^30–35^. HG beams are typically generated using phase plates^36^ and they can also be found in applications such as optical traps for atoms^37^, near field optical microscopy^38^, and optical wireless communication to add robustness to optical links in turbulent conditions^39^.

In this work it is proposed to monolithically integrate optical amplifiers in the circuitry through an InP generic platform. The integrated photonic platform embeds both passive building blocks and active ones such as semiconductor optical amplifiers (SOAs) and photodetectors that operate in a wide wavelength range (up to 150 nm 3 dB bandwidth of net modal gain was demonstrated in previous work^40,41^). Tunable lasers can also be integrated on the same optical chip, thus eliminating the losses associated with fiber-to-chip coupling. In the InP OPA PIC presented here we realise an integrated 8-arms OPA with a booster amplifier and independently controlled SOAs and phase modulators in each arm and for the first time, to the best of our knowledge, we measure the amplification level through the OPA and its output power over a wide wavelength range of operation. Beam forming and steering are demonstrated over the same wavelength range. Furthermore, utilizing the independently controlled SOAs and phase modulators in the OPA arms, we demonstrate the programmable beam shaping of the emitter power profile. This capability was used to minimize the diffraction sidelobes by using a Gaussian power distribution over the arms, and to demonstrate the generation of Hermite–Gaussian (HG) beams up to the second order.

In the following sections, we describe the PIC design and characterization results, the outcome of which is reported in Table 1. The SOAs on the chip generate heat which often limits the overall output power performance of the array. We present an analysis of the thermal crosstalk between neighboring arms of the OPA. This is followed by a description of the results of calibration and beam steering. Finally, through the active control of the amplitude and phase in the OPA arms, we show the shaping of the OPA emitter power distribution to reach high SLSR levels, and to form free-space HG beams.

**Table 1:** Optical phased array specifications in state of the art, where on-chip losses and optical power levels are reported. The table entries are sorted from high to low optical losses in the OPA circuits. [*] Power in free-space, at the output of the OPA emitter, divided by input power coupled into the PIC. The fiber coupling losses are not considered here. [**] Estimated. [^▿^] Power in the main lobe. [^†^] Fiber-coupling loss was not reported. .

Ref.	Platform	Numb. antennas	SLSR, dB	Wavelength, nm	Max power out, mW	Loss or gain, dB *
^23^	Si	64	N/A	1502	$32 \cdot 10^{-6}$ ^▿^	−43.0
^19^	Si	64	10.4	1525–1570	0.140	−31.8
^45^	Si	64	8.8	1550	0.004	−29.0
^16^	SiN-Si/InP	256	11.0	1472–1571	6	−17.0
^12^	SiN-Si	128	10.3	1350–1630	N/A	−15.5
^46^	InP	32	8.2	1550	N/A	−12.9
^13^	InP	100	6.3	1550	N/A	−12 $\sim$ -15 [**]
^47^	Si	24	7.0	1540–1560	N/A	−11.2
^10^	Si	8192	10.0	1510–1570	400	−11 ^†^
^15^	SiN-Si	256	10.8	1510–1630	0.025 ^▿^	−10.6
^17^	SiN-Si	64	9.9	1540–1630	0.13 ^▿^	−10.1
^21^	SiN-Si	32	N/A	1500–1600	14	−10.0 ^†^
^48^	SiN	16	11.5	1525	0.005	−8.3
^49^	SiN-poly	64	N/A	1550	N/A	−8.2
^20^	Si	32	9.3	1530–1610	N/A	−8.1
This work	InP	8	19.8	1465–1590	35.5	+21.5

## Circuit design of the optical phased array

**Figure 1:**
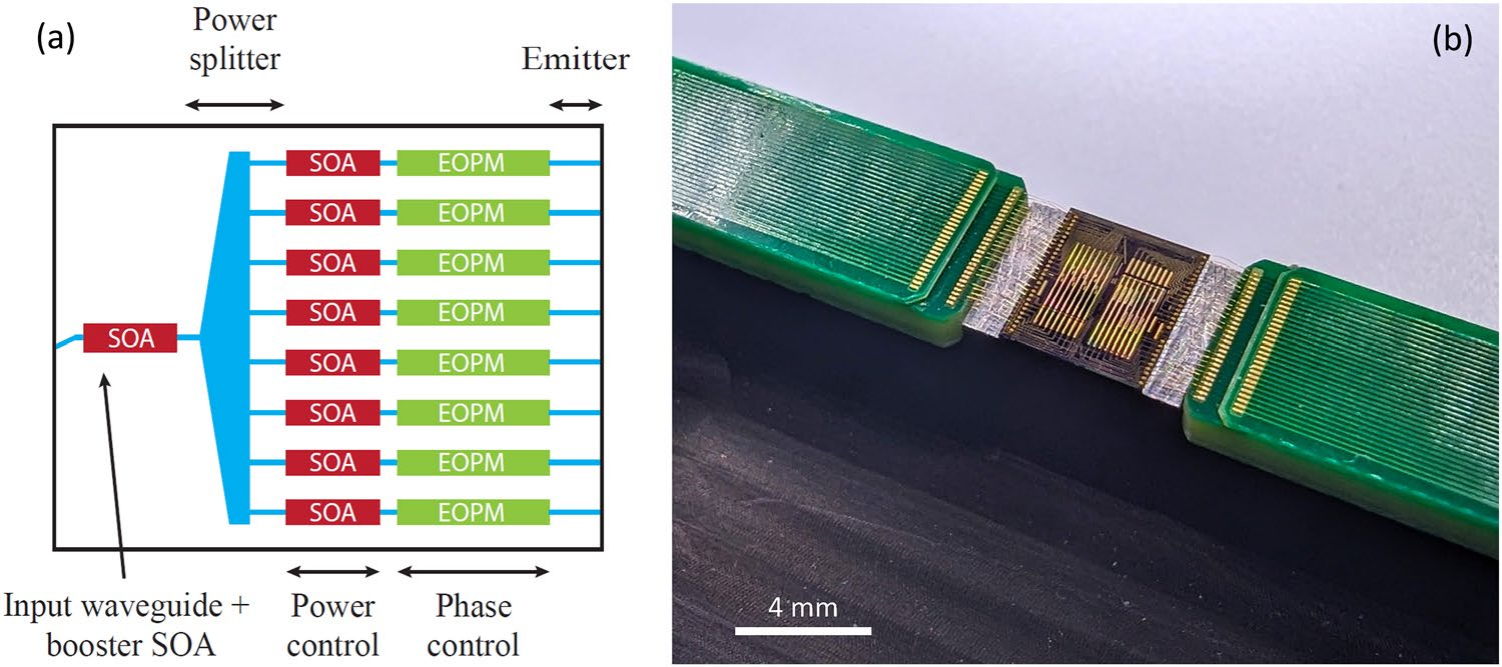
(**a**) OPA circuit design schematic (not to scale). (**b**) Photograph of the OPA assembly showing the PIC on an Aluminium mount and wire-bonded to PCBs on each side. The chip contains two OPA PICs; the one used in this work is shown in the left half of the chip.

A schematic of the designed OPA PIC is shown in Fig. 1a. The input of the circuit consists of a waveguide optical port (angled to reduce reflections at the PIC facet) for fiber coupling of an external tunable laser, and a 500 $\mu$m long booster SOA. The light is split into 8 equivalent arms through a star coupler with a Gaussian power distribution over its output waveguides; deep etched arrayed waveguides of 1.5 $\mu$m width were used. To minimize the splitter losses we limited the spacing between the array waveguides to minimum reliable manufacturable levels (200 nm). We optimized the radius of the star coupler through *Lumerical varFDTD* simulations to achieve a Gaussian distribution power ratio of 6 dB (i.e., the power ratio between the inner and outer arrayed waveguides). This results in star coupler losses of 0.3 dB, and 20 dB SLSR at the output. To achieve further amplification of the input light, each OPA arm consists of a 500 $\mu$m long SOA. The programmable shaping of the OPA emitter power profile is achieved by independently driven amplifiers in the circuit. Each arm amplifier is followed by an electro-optical phase modulator (EOPM, 2.2 mm long) to control the phase profile of the OPA emitter allowing both shaping and steering of the generate beams. The EOPMs operate under reverse bias and have leakage currents below 500 $\mu$A, therefore having a negligible power consumption.

The OPA emits light in free space through a uniformly distributed array of edge emitting waveguides (1.2 $\mu$m wide, 2.2 $\mu$m pitch). Their positions and widths were optimized through *Lumerical FDE* and *FDTD* simulations to achieve negligible optical coupling between adjacent emitter waveguides and to obtain beam scanning in a field of view (FoV) of $\pm 20.5^\circ$, ensuring the presence of only the zero and first order grating lobes at all steering directions. The PIC’s optical facets have an anti-reflection coating to reduce the reflections at the facet and avoid lasing in the OPA circuit. The OPA was fabricated on an InP generic platform^41,42^ through a multi-project wafer run by *SMART Photonics*; the assembled PIC is shown in Fig. 1b.

## On-chip amplification and beam steering in a wide wavelength range

We measured the optical gain of the OPA for wavelengths between 1465 nm and 1600 nm (in 5 nm steps), for a range of input power levels (−20 dBm to 8.5 dBm) and SOA driving conditions (3–10 kA/cm^2^). Unless otherwise specified, when we mention the input power throughout this article we always refer to on-chip levels (i.e., power after fiber-coupling losses); the output power refers to the total optical output of the OPA, considering grating diffraction orders and sidelobes. The net on-chip gain is defined as the difference between the absolute SOA gain and the insertion losses of passive components in the circuit, and it is measured as the difference between the input and output power levels.

**Figure 2:**
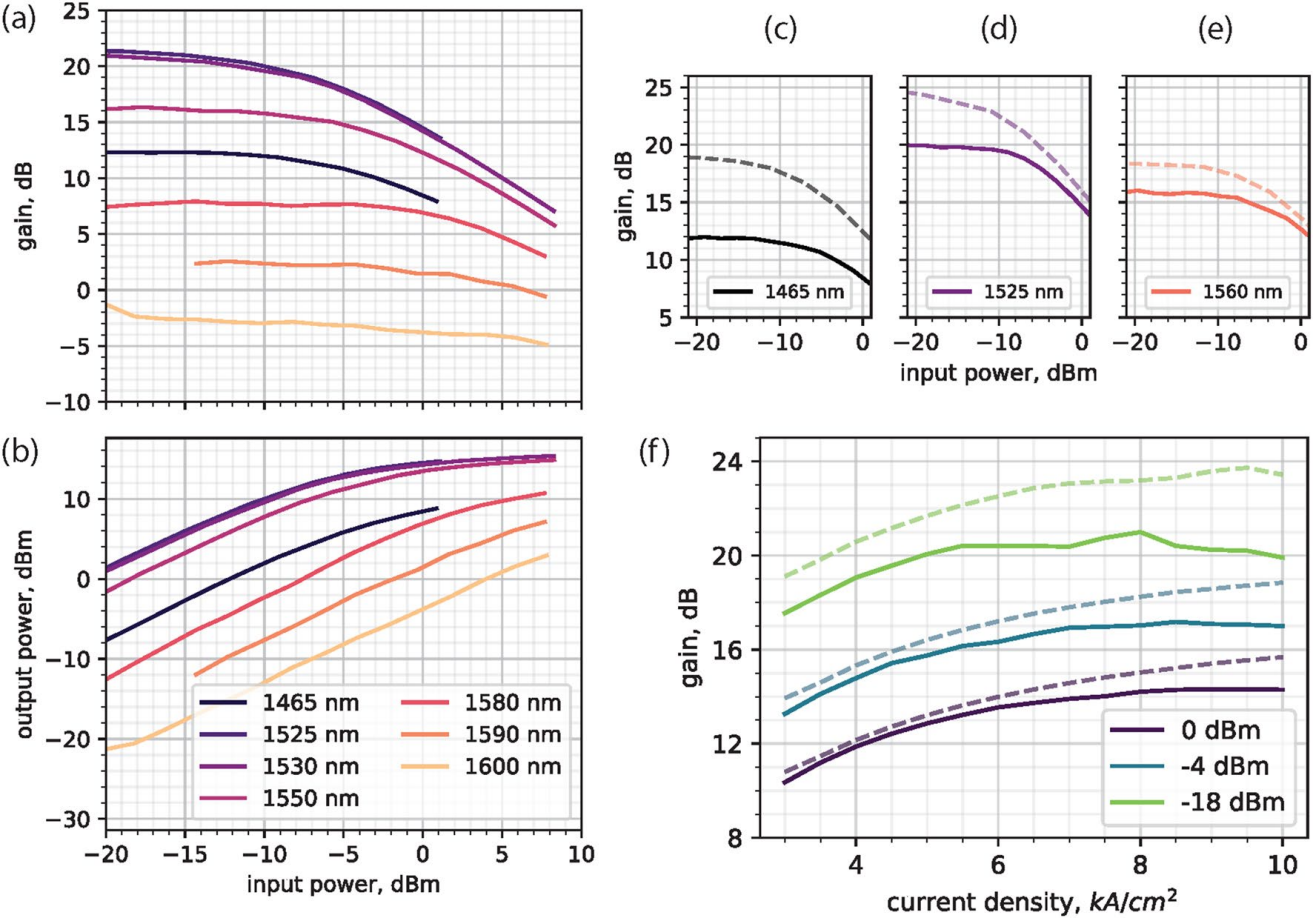
(**a**) Measured net gain of the OPA, and (**b**) total output power level at the output facet of the PIC as a function of on-chip input power. The booster SOA and arrayed SOAs were driven at 10 kA/cm^2^ and 7.5 kA/cm^2^ respectively. The legend in (**b**) is also valid for (**a**). Not all wavelengths in the sweep are shown for clarity purposes. (**c**–**f**) Comparison of gain measured through the arrayed SOAs (solid lines) and with consecutively driven SOAs (dashed lines). In (**c**–**e**) the SOAs are driven at 10 kA/cm^2^. In (**f**) the tunable laser wavelength is set to 1530 nm and the current density is swept to understand gain deterioration due to thermal effects.

Figure 2a, b shows the net on-chip gain and the output power for a maximum current injection of the booster (10 kA/cm^2^) and for a 7.5 kA/cm^2^ current injection of all arrayed SOAs. This driving condition was selected to limit the degradation of gain due to the mutual thermal influence of neighboring SOAs (thermal crosstalk). Under the aforementioned current driving conditions, and for on-chip input power levels as low as −20 dBm, we measured a maximum gain of 21.5 dB at the 1525 nm wavelength which, to the best of our knowledge, is the highest gain measured through an OPA. The maximum wavelength of 1525 nm, indicates a red-shift with respect to the measured gain spectrum of a single SOA^40^ [Supplementary Figure S2], and we attribute this to the extra heat generated by all the SOAs in the circuit, compared to a self-heating single SOA. For input power levels as high as 6 dBm, the net on-chip gain is positive for more than 125 nm, which is comparable with the wavelength range of operation for OPAs in the state of the art. For shorter wavelengths, this range is limited by the available measurement instrumentation (tunable laser source). The maximum measured power emitted from the chip, 15.5 dBm (35.5 mW), was obtained with 8.5 dBm input power (Fig. 2b) and 1530 nm wavelength, which drops by 3 dB in a 40 nm range (limited by instrumentation). This, to the best of our knowledge, is the highest output power level recorded through an integrated OPA with on-chip amplification.

### Thermal investigation of the OPA performance

The performance of the SOA optical gain as a function of current injection is shown in Fig. 2f (dashed lines). For large current densities, we observe a roll-over of the gain due to thermal crosstalk of neighboring SOAs in the OPA arms. The heat generated by continuously driven SOAs at large current densities, and the proximity of SOAs in the arms (160 $\mu$m spacing between them) ultimately limit the maximum gain of the SOAs in the array.

We have studied the influence of thermal crosstalk on the OPA gain as a function of current density and input power level by comparing the gain measured in two ways. The first measurement was as described in the previous section: all the arrayed SOAs are simultaneously and continuously driven, thereby thermally influencing one another. With the second measurement, we drive one arrayed SOA in forward bias, and use the other 7 SOAs to absorb light by applying a reverse bias voltage ($-4$ V). In this way, the output power contribution from each OPA arm could be measured, while the mutual thermal crosstalk between SOAs was reduced. With an input power of 0 dBm, we measured up to 1 mA of photocurrent in the reverse biased central SOAs and 0.25 mA in the outer ones, giving up to 4 mW power dissipation. For the forward biased SOA, the current density was swept between 3 and 10 kA/cm^2^. For the 2 $\mu$m wide shallow waveguides that support the SOAs, this translates to current levels in the 30–100 mA. The measured voltage between 1.2 and 2.2 V results in a worst-case power dissipation of 36–220 mW. Thus for the range of input power and current densities considered in this study (Fig. 2c–f), the power dissipated by the forward biased SOA is at least 4.5 times higher (and typically much higher in the other driving conditions) than the one dissipated by two neighboring reverse biased SOAs. By consecutively measuring the output power though all of the independently driven OPA arms and summing their contribution, we obtain an estimate of the total output power, and hence OPA gain, when the mutual thermal interaction between adjacent SOAs is minimized. The method and its schematic representation are shown in the supplementary material through [Supplementary Figure S3].

A comparison of the OPA gain obtained with the two types of measurements is shown in Fig. 2c–f. Here the solid lines represent the 8 SOAs driven simultaneously, and the dashed lines represent the sum of contribution from independently driven SOAs. By reducing the thermal crosstalk to a minimum, we can demonstrate up to 3.5 dB higher gain compared to the results discussed previously.

To further support the thermal investigation of this OPA circuit design, we carried out a thermal simulation (COMSOL, [Supplementary Figure S4, S5]). We estimate that the average temperature increase in the SOA core due to the thermal crosstalk with neighboring arms is 6 ^∘^C at the maximum current driving condition of 10 kA/cm^2^. Moreover, through the modal gain characterization of the SOA at different temperatures [Supplementary Figure S6], we estimate that for a 500 $\mu$m long SOA, such temperature variation corresponds to $\sim$2 dB gain difference at 1525 nm. Considering the accuracy limits of the measurement and simulation techniques, this is in accordance with the measured gain drop shown in Fig. 2c–f. For simplicity, the booster SOA’s thermal influence was not considered in the thermal simulations; however, we expect a further drop in gain due to additional rise in temperature.

### Beam steering

In this section we demonstrate the beam steering operation in the broad wavelength range where the OPA gain was measured (1465–1600 nm). Through the setup shown in Fig. 6 and described in the Methods section, we carried out the OPA calibration in the far-field using the modified rotating element electric field vector (mREV) method, as demonstrated in our previous work^43^. The calibration routine returns a set of reverse bias voltages that, when applied to the EOPMs, allow the OPA to form a beam at the calibration angle in the far-field. Moreover, the phase-voltage relation for each EOPM is extracted with this method, which makes it possible to steer the main diffraction order to any angle. In the wavelength range of interest, the reverse bias voltages applied to the EOPMs to achieve $2\pi$ phase shift are in the 6.4 V to 9.1 V range [Supplementary Figure S7]. The reverse bias voltages applied to the EOPMs during the calibration routine were in the 0 - 10 V range, with 0.2 V steps. The booster and arrayed SOAs were operated with current densities of 10 kA/cm^2^ and 7.5 kA/cm^2^ respectively. We employed the maximum input power level (shown in Fig. 2 a,b) for all wavelengths to achieve the highest suppression between the ASE level and the beam peak level.

**Figure 3:**
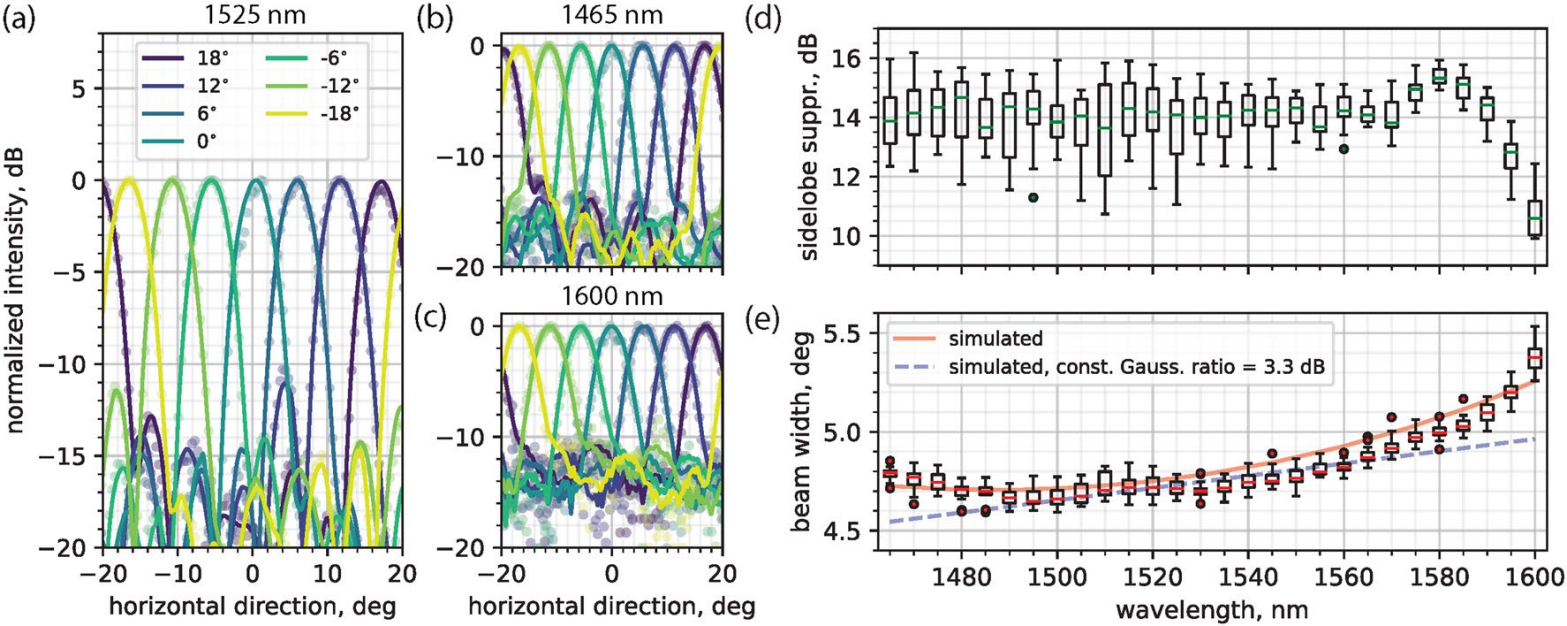
(**a**–**c**) 1D cross section of the far-field beam at different steering directions within the FoV at (**a**) 1525 nm, (**b**) 1465 nm, and (**c**) 1600 nm wavelengths. (**d**) Measured sidelobe suppression ratio (SLSR). For each wavelength, the median value across different steering angles is shown inside the box plots. (**e**) Measured beam width (FWHM, box plots). The dashed blue curve shows the simulated beam width for a fixed Gaussian distribution power ratio of 3.3 dB. The solid red curve represents the simulated beam width for a wavelength-dependent Gaussian distribution power ratio.

The results of calibration and steering are shown Fig. 3. We show beam steering within the $\pm 20.5^\circ$ field of view with a full-width at half-maximum (FWHM) in the $4.6^\circ - 5.5^\circ$ range across the steering and wavelength range. The FWHM is expected to grow linearly with the wavelength due to diffraction, as it’s shown in the far-field simulation result (*Lumerical FDTD*) of Fig. 3e (dashed blue curve), where we used a constant Gaussian distribution power ratio value of 3.3 dB. This value was measured through near-field imaging at 1525 nm, however this is not constant across the wavelength range of interest as it is shown in [Supplementary Figure S8, S9]. Because of this wavelength dependence, the beam width measurement results deviate from the linear relation for wavelengths that are longer and shorter than the OPA gain spectrum’s central wavelength (i.e., 1525 nm). We attribute the variation of Gaussian distribution power ratio with wavelength to the sub-linear regime of the amplifiers at high input power levels. In fact, because of the 6 dB Gaussian distribution given by the star coupler, the arrayed SOAs operate at different sub-linear regimes. As a result, the Gaussian distribution power ratio is compressed at the output of the amplifiers stage. For wavelengths that are longer and shorter than 1525 nm (at the gain peak), the booster amplification level is lower, thus moving towards the linear operation regime of the arrayed amplifiers. This results in a larger value of the power ratio between the inner and outer OPA arms, which in turn broadens the far-field beam width^18,44^. This effect was further investigated by running simulations with wavelength-dependent Gaussian power distribution values, which were measured through near-field imaging at different wavelengths [Supplementary Figures S8–S11]. The comparison between the simulation results for the beam widths (Fig. 3e, solid red curve) and the measured ones shows a good match both in the wavelength trends and the beam width values.

We have also recorded the far-field sidelobe suppression ratio (SLSR, measured as the power ratio in dB between the peak intensity of the main grating order and the highest intensity diffraction sidelobe) at all wavelengths and steering angles. The median SLSR is above 13.6 dB, with up to 1.3 dB increase, in the 1465–1575 nm wavelength range. The measurement results show lower SLSR values compared to the simulated ones [Supplementary Figure S13]. This is attributed to the SOAs amplified spontaneous emission (ASE) which is also collected by the imaging system and projected onto the camera sensor. The ASE increases the background level and leads to a lower measured SLSR. This effect is strongest for the longest wavelengths under study. At those wavelengths the gain efficiency drops and the ASE level increases and becomes comparable to the signal strength. As a consequence the median of the measured SLSR values drops down to 10.6 dB. Furthermore, we attribute the variation of SLSR across different wavelengths and steering angles to the aforementioned wavelength-dependent compression of the Gaussian distribution power ratio, while the undesired electro-absorption of the EOPMs driven at different voltages^43^ adds power fluctuations in the OPA arms that distort the Gaussian power distribution given by the star coupler. Both effects are responsible for a deterioration of the SLSR.

## Programmable beam shaping through amplitude and phase control

As we mentioned in the previous sections, having independently driven SOAs in the OPA is beneficial to generate a free-space beam with higher power which, in the case of LiDAR sensing, translates into a longer detection range. This performance improvement comes at the cost of an increased complexity in the electrical driving of the circuit. However, the independent driving of amplifiers offers versatility to the OPA beam forming. In this section, we present the results of a programmable beam shaping through the amplitude and phase control in the OPA arms.

### Sidelobe suppression through shaping of the emitter power distribution

**Figure 4:**
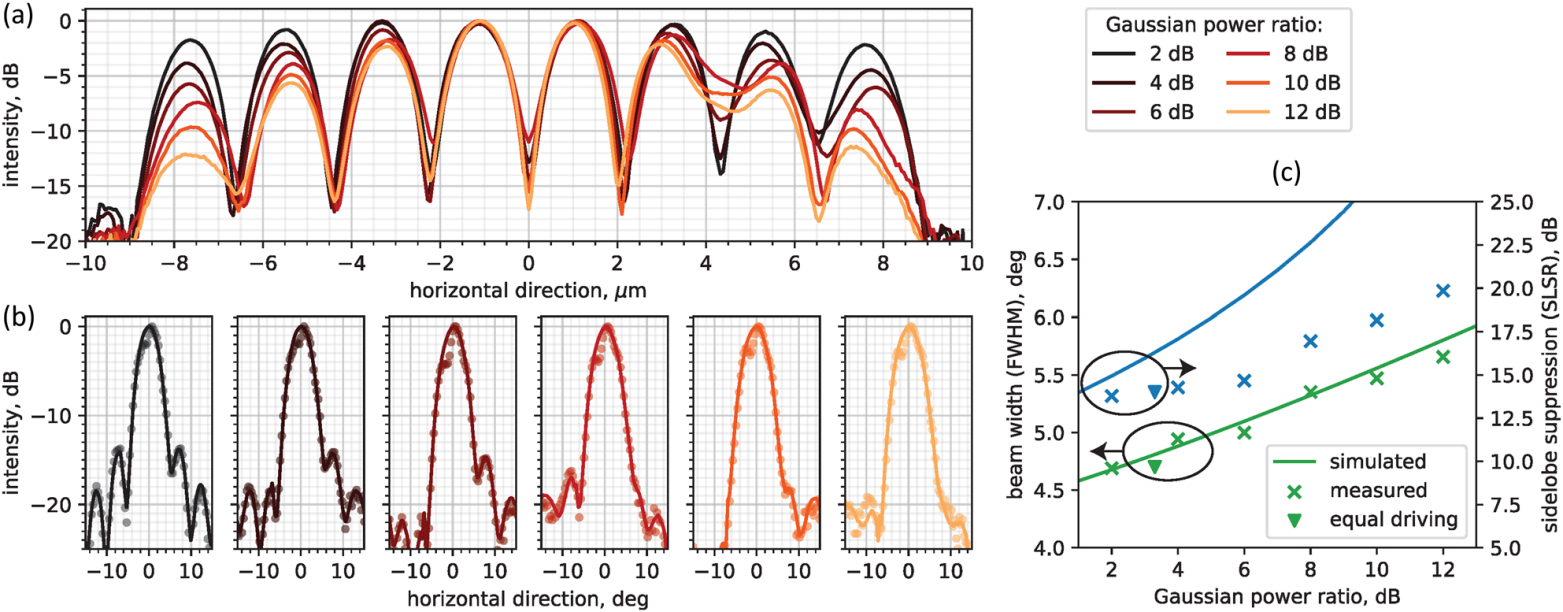
(**a**) 1D near-field profile of the OPA emitter. The power ratio between inner and outer arms is swept from 2 dB to 12 dB. (**b**) Main far-field lobe (steering at 0^∘^) and (**c**) beam properties for different Gaussian distribution power ratio values. The crosses and solid lines represent the measurement data and simulation results respectively. The triangles represent the measured FWHM and SLSR when the arrayed SOAs are driven with the same current density of 7.5 kA/cm^2^. All measurements in this figure were performed at 1530 nm wavelength.

To display the capability of the OPA with independently driven amplifiers, we experimentally demonstrated an improvement of the beam in the far-field through the suppression of the diffraction sidelobes. This was achieved by varying the Gaussian power profile of the OPA emitter through the control of the SOAs current levels. The shape of the Gaussian profile was modified by varying its inner to outer arms power ratio; the waist of the Gaussian profile is derived accordingly, assuming a symmetric distribution with respect to the center of the emitter. An increase in the Gaussian distribution power ratio corresponds to an increase in the sidelobe suppression ratio SLSR^18^, as it is shown in Fig. 4.

We applied the Gaussian power apodization by sweeping the currents in the arrayed SOAs, until the measured near-field intensity profile matches the theoretical one. The procedure is repeated for each OPA arm. The current density in each SOA is swept using 0.2 kA/cm^2^ steps, in the 3–6 kA/cm^2^ range. The measurements were performed with 8 dBm on-chip input power at 1530 nm, and 10 kA/cm^2^ booster SOA driving current density. After the optimization of the power profile, we calibrated the phase distribution in the OPA arms following the mREV method, to form a beam at 0^∘^. The EOPMs reverse bias voltages were swept in the 0 - 10 V range, with 0.2 V steps. By applying different values of the Gaussian distribution power ratio to the near-field profile, we demonstrated up to 19.8 dB SLSR in the far-field main lobe. This is 10 dB higher than the typical values reported in literature (Table 1) and, to the best of our knowledge, it is the highest SLSR recorded in OPAs with embedded amplifiers (i.e., where the amplifiers spontaneous emission affects the SLSR measurement).

Furthermore, we compared the measured beam properties (FWHM and SLSR) with the ones we simulated (*Lumerical FDTD*). The measured FWHM matches well with the simulated values, showing that a 10 dB increase of the Gaussian distribution power ratio brings a 21% increase in beam width, whereas the measured SLSR is close to the simulated one for low power ratio levels, but diverges significantly at larger levels. We attribute this disparity to the limitations of the measurement system (i.e., limited angular resolution of $0.37^{\circ }$ per pixel and accuracy of the phase calibration method), to the camera background level given by the SOA’s amplified spontaneous emission, and to the power variations in the OPA arms during calibration due to voltage-dependent absorption in the EOPMs^43^ which creates a mismatch between the ideal near-field Gaussian profile and the measured one. The beam properties calculated for the beam steering measurement (i.e., median beam width and SLSR at 1530 nm from Fig. 3) are also reported in Fig. 4c (triangle symbols) as a reference, as well as the measured near-field Gaussian distribution power ratio (3.3 dB).

### Generation of 1D Hermite–Gauss beams

**Figure 5:**
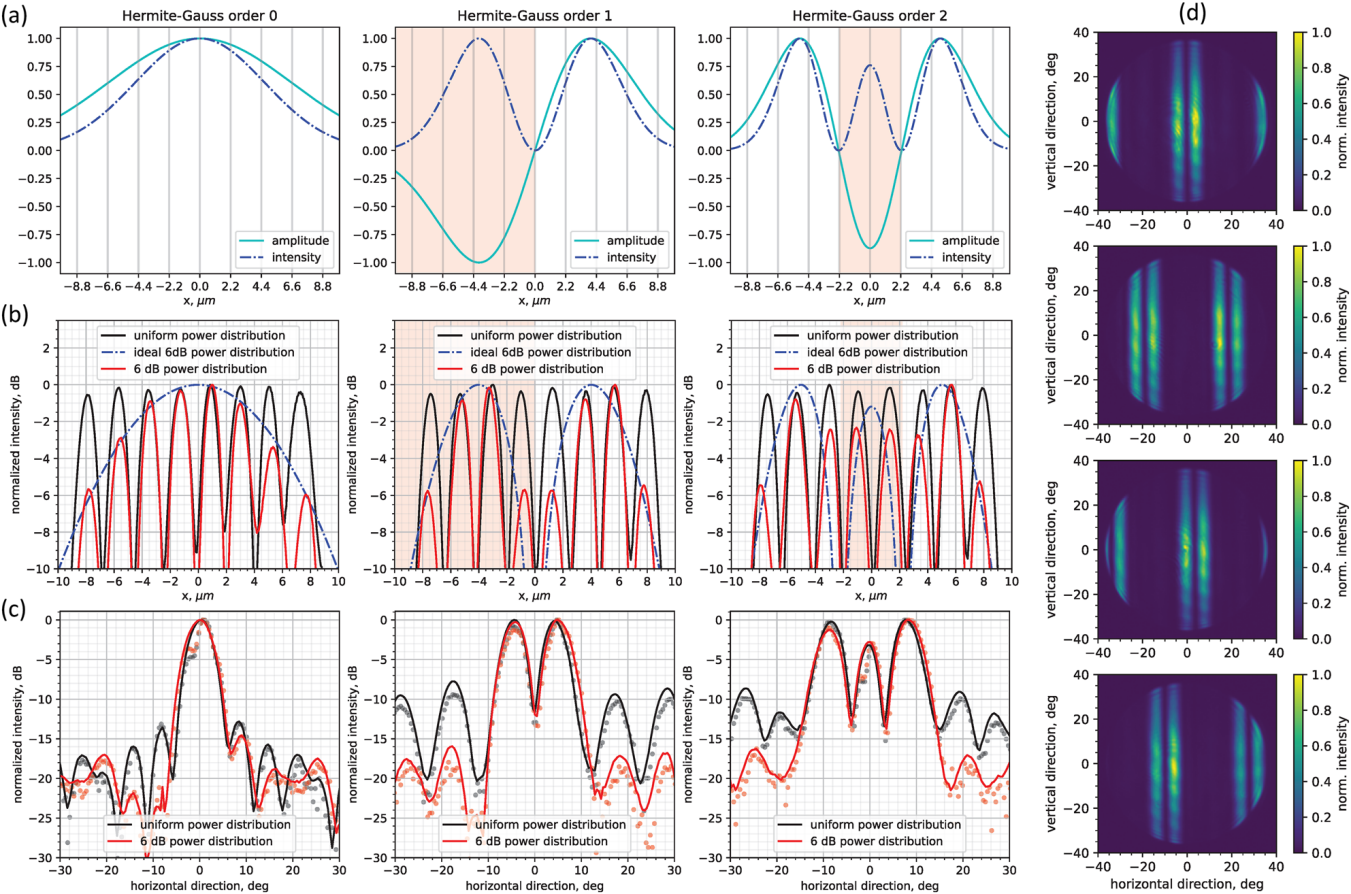
(**a**) Theoretical Hermite–Gauss (0, 1, 2 order) amplitude (*E*-field) and intensity ($|E|^2$) profiles. The portion of profile with negative amplitude is highlighted in red. Near-field (**b**) and far-field (**c**) images of the OPA with different power distributions in the arms, following orders 0, 1, and 2 Hermite–Gauss beams. The black plots represent HG beams where the targeted power distribution is uniform. Red plots represent HG beams where both amplitude and phase in the OPA arms are controlled to achieve the desired HG beam. The near-field emitter waveguide modes where a $\pi$ phase shift is applied to achieve negative amplitude are highlighted in red. (**d**) Beam steering of the HG1 beam at four angular positions.

The independent control of phase and amplitude in all arms of the OPA was exploited to generate 1D beams with more complex shapes. As an example of such capability we demonstrated the generation of 0th, 1st, and 2nd order Hermite–Gauss (HG) beams (Fig. 5). We employed the procedure described in the previous section to sample and replicate the HG beam’s intensity distribution (Fig. 5a) to the power profile of the OPA emitter (red curves in Fig. 5b) through the tuning of the arrayed SOAs currents. The sign in the HG beam amplitude profile was replicated through 0 and $\pi$ phase shifts in the OPA arms given by the EOPMs after the phase calibration routine.

The resulting far-field profiles (Fig. 5c) are one-dimensional quasi-HG modes which are elongated in the vertical direction due to the aspect ratio of the OPA emitter, and periodically reproduced at $40^\circ$ angular intervals due to the grating nature of the emitter array with spacing larger than $\lambda /2$. We demonstrated beam steering within the field-of-view range for the 1st order HG mode, which is shown in Fig. 5d. The ability to control both amplitude and phase in all OPA arms offers an interesting level of flexibility when it comes to generating beam shapes with a high sidelobe suppression level. In fact, as Fig. 5c shows, we demonstrated that controlling both the amplitude and the phase to match the theoretical HG near-field profiles generated far-field HG-0, HG-1, and HG-2 beams with more than 15 dB SLSR, compared to SLSR levels as high as 10 dB in the case of HG beams generated by a uniform power profile.

## Conclusions

We have demonstrated the first InP OPA with on-chip amplification reaching up to 21.5 dB net gain and up to 35.5 mW output power, while being able to operate in a 125 nm wavelength range. The achieved power levels meet the requirements for LiDAR coherent sensing at several tens of meter, and the achieved wavelength range is highly relevant to provide flexibility in the design of multi-wavelength LiDAR architectures. Moreover, we performed a study of the thermal limitations in the gain of the SOA array, showing a potential gain improvement of 3.5 dB if thermal cosstalk is minimized. Finally, we demonstrated the programmable beam shaping of the OPA beam thanks to the simultaneous and independent control of the amplitude and phase in the OPA arms. We demonstrated up to 19.8 dB sidelobe suppression ratio by tuning the power ratio of the OPA emitter’s Gaussian distribution of the output waveguides and we showed the generation of 0th, 1st, and 2nd order Hermite–Gauss beams and their steering in free space with large (> 10 dB) SLSR. The results show the versatility of OPA designs with on-chip amplification for customized beam shaping, which may find immediate applications in LiDAR or OWC systems.

## Methods

**Figure 6:**
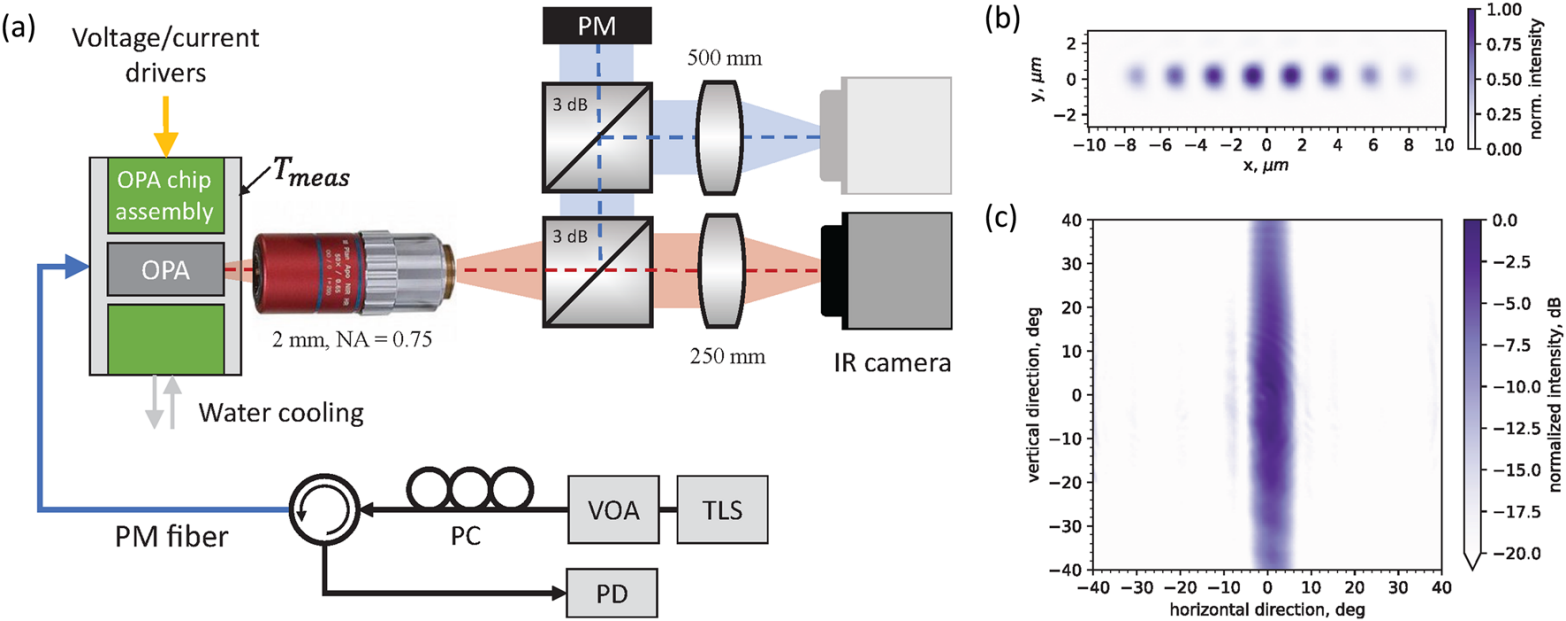
(**a**) Schematic diagram of the setup employed to characterize the gain and optical properties of the OPA. Near-field (**b**) and far-field (**c**) images at 1525 nm wavelength and $0^\circ$ steering.

We measured the output power emitted in free space, and we performed optical characterization of the OPA’s near-field and far-field through the setup shown in Fig. 6. The OPA’s net on-chip gain was calculated as the difference between the on-chip input power and the measured output power. The on-chip input power level was measured through the booster SOA operated under reverse bias condition, whose responsivity (i.e., the conversion of photons in photocurrent, measured in A/W) is provided by the foundry.

The output radiation of the chip was collimated by a microscope objective lens (numerical aperture $NA=0.7$, focal length $f = 2$ mm), and then split into three optical paths through two cascaded 3 dB splitters. The OPA’s near-field and far-field are then focused on an IR camera (*Allied Vision Goldeye G-033*) through plano-convex lenses ($f_1 = 250$ mm, $f_2 = 500$ mm) placed in two of the beam’s optical paths. The angular resolution of the far-field imaging system is $0.37^\circ$ per pixel, and the beam widths shown in Figs. 3 and 4 were calculated by interpolating the camera data points in a 1D cross section of the far-field image. The third beam is collected by an integrating sphere powermeter (*Newport 818-IR*) to measure the output power of the OPA. A 13.3 dB free-space loss between the output of the OPA and the powermeter was recorded by measuring the power directly in front of the OPA. The lasers (*Quantifi Photonics TLS-1004/1005, Agilent 81980A*) input power levels were swept through a variable optical attenuator followed by a polarization controller, to keep a fixed state of polarization (TE). The input power level, after fiber and instrument losses, was characterized by reverse-biasing the booster amplifier and measuring the photocurrent flowing through it. The chip was glued (EPO-TEK H20E epoxy, with 29 W/mK thermal conductivity) to an Aluminium assembly unit, which was water cooled at a constant water temperature of 18 ^∘^C. The chip substrate temperature was monitored by a thermistor placed in the Aluminium block, underneath the PIC. Moreover, the PIC assembly is wire-bonded to two printed circuit boards (PCB) which routed the wire-bonded PIC connections to the voltage and current drivers (*Nicslab XDAC120*). Chip alignment to free space and fiber alignment to the OPA input waveguide were performed before each measurement using the on-chip light sources to track light coupling variations. A circulator routes the light generated by the chip to a photodetector for this purpose.

### Supplementary Information

Supplementary Information.

## Data Availability

The datasets generated during and/or analysed during the current study are available from the corresponding author on reasonable request.
