# Supplementary material for: Integrated optical phased array with on-chip amplification enabling programmable beam shaping
Source: Sci Rep. 2024 Apr 26;14:9590. doi: 10.1038/s41598-024-60204-5 (PMC11053068; doi:10.1038/s41598-024-60204-5)
Supplement: Supplementary file 1 — Supplementary Information. [file 41598_2024_60204_MOESM1_ESM.pdf]

Integrated optical phased array with on-chip amplification enabling programmable beam shaping

Supplementary Information

Marco Gagino[1,*], Alonso Millan-Mejia[2], Luc Augustin[2], Kevin Williams[1], Erwin Bente[1], and Victor Dolores-Calzadilla[1]

[1]Technical University of Eindhoven, Eindhoven Hendrik Casimir Institute, Eindhoven, 5612 AP, The Netherlands
[2]SMART Photonics, Eindhoven, 5656 AE, The Netherlands
[*]m.gagino@tue.nl

Contents

1. **Circuit design of optical phased array** 2
2. **Modal gain of the SOA** 3
3. **Thermal aspects of the SOA array** 4
4. **Calibration and EOPM characterization** 8
5. **Near-field measurement of the OPA Gaussian distribution power ratio** 9
6. **Simulations of far-field properties** 11

1. Circuit design of optical phased array

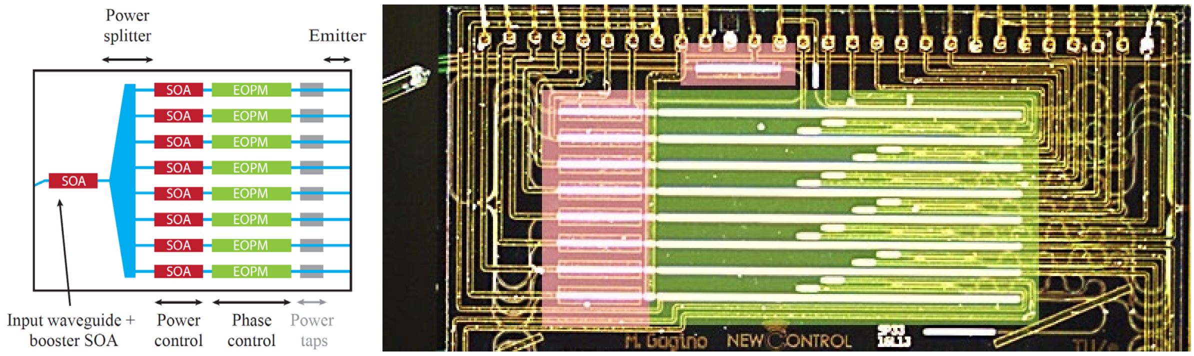

Supplementary Figure S1. Schematic view (left) and microscope photograph (right) of the designed and fabricated OPA. The 8 arrayed amplifiers and the booster SOA are highlighted in red. In green, we highlighted the 8 electro-optical phase modulators that tune the phase in the 8 OPA arms.

The microscope image in Supplementary Figure S1 shows the fabricated photonic integrated circuit (PIC) containing the optical phased array (OPA) used in this publication. More information about the geometrical specification of the OPA's building blocks is provided in the following Supplementary Table.

Supplementary Table S1. Design properties of the OPA with amplification

**Building block**	**Properties**	
Booster SOA	Length of amplifier	$500\ \mu m$
Arrayed SOAs	Length of amplifier	$500\ \mu m$
	Spacing	$160\ \mu m$
Star coupler	Gaussian distribution power ratio	$6\ dB$
Electro-optical phase modulators	Length of modulator	$2.2\ mm$
Emitters array	Spacing of waveguides	$2.2\ \mu m$

The waveguide propagation losses were measured by the foundry (SMART Photonics) through dedicated test dies that were manufactured on the same wafer. The characterization through the Fabry-Perot method [i] resulted in propagation loss for the standard ($1.5\ \mu m$ wide) deep-etched waveguides of $3.12\ dB/cm$, with a $0.26\ dB/cm$ standard deviation. The measured losses for deep-etched waveguide bends with $100\ \mu m$ radius used in the OPA circuit is $0.066\ dB/90°$, with a $0.008\ dB/90°$ standard deviation.

Besides the devices mentioned in the main text of this article, each arm embeds a power tap for on-chip power monitoring, the use of which is out of scope for this publication. The power tap consists of two cascaded 85/15 imbalanced multi-mode interferometers (MMIs), each of which routes some of the power (15%) to a region with photodetectors. We estimate that at least 1.4 dB of the total OPA loss comes from the power taps, as calculated from the splitting ratio of the two cascaded 85/15 MMIs. The actual total loss may be higher due to the insertion losses in the MMIs.

2. Modal gain of the SOA

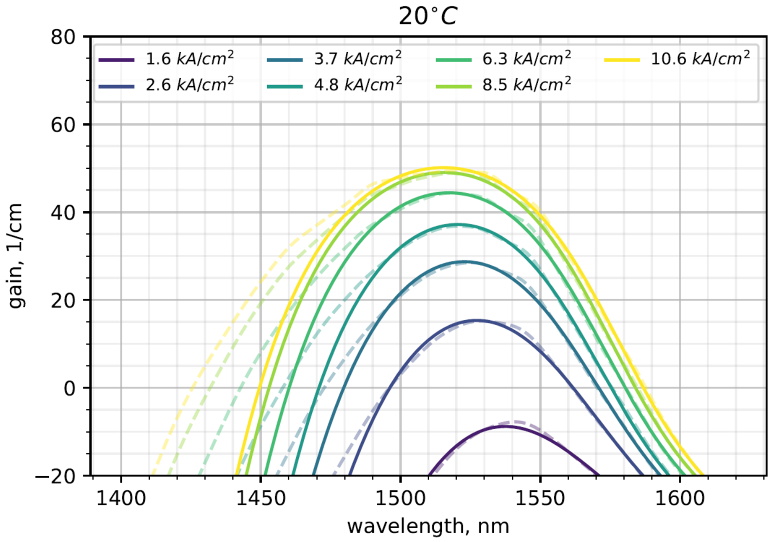

Supplementary Figure S2. Modal gain spectrum measured at different driving current densities (top, measured at $20°C$ substrate temperature). The dashed lines are measurement data; the solid lines are fitted curves.

The Supplementary Figure shows the modal gain spectrum of an SOA. The measurements of the amplifier performance were done using a separate chip containing test structures for individual building block characterization. We employed the multi-section SOA characterization method described in [ii] to measure and fit the modal gain for different biasing current densities and substrate temperature. The results of the modal gain reported in this Supplementary Figure are obtained under the assumption of zero losses in between the multiple SOA sections, where electrical isolation waveguide sections are placed to prevent electrical shorts.

3. Thermal aspects of the SOA array

OPA gain measurement without thermal crosstalk

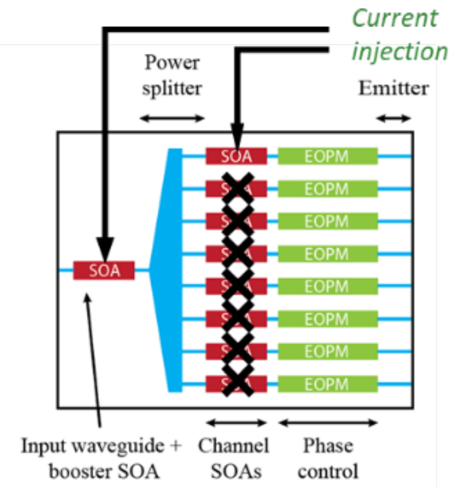

Supplementary Figure S3. Schematic of the method used to characterize the OPA gain while minimizing the thermal crosstalk between neighboring arms. The crossed out SOAs were biased with a -4V reverse bias voltage to absorb light and prevent light transmission into the free-space. The emission of a single arm is measured one at a time.

The above schematic (Supplementary Figure S3) shows the method used to characterize the OPA net gain with no thermal crosstalk effects between neighboring arms. The booster SOA is kept at a constant biasing condition. One by one, each arrayed SOA is forward biased through current injection, while the other 7 SOAs are reverse biased to absorb light. The output power from each OPA arm is measured through the free-space powermeter as described in the Methods section of this publication. Once the amplified spontaneous emission component of the measured power is removed from the power measurement of each arm, the total output power is calculated by summing the 8 arms contributions. The net OPA gain measured through this technique is reported in Figure 2 (c-f) of the main publication.

Thermal simulation of a single SOA and of a SOA array

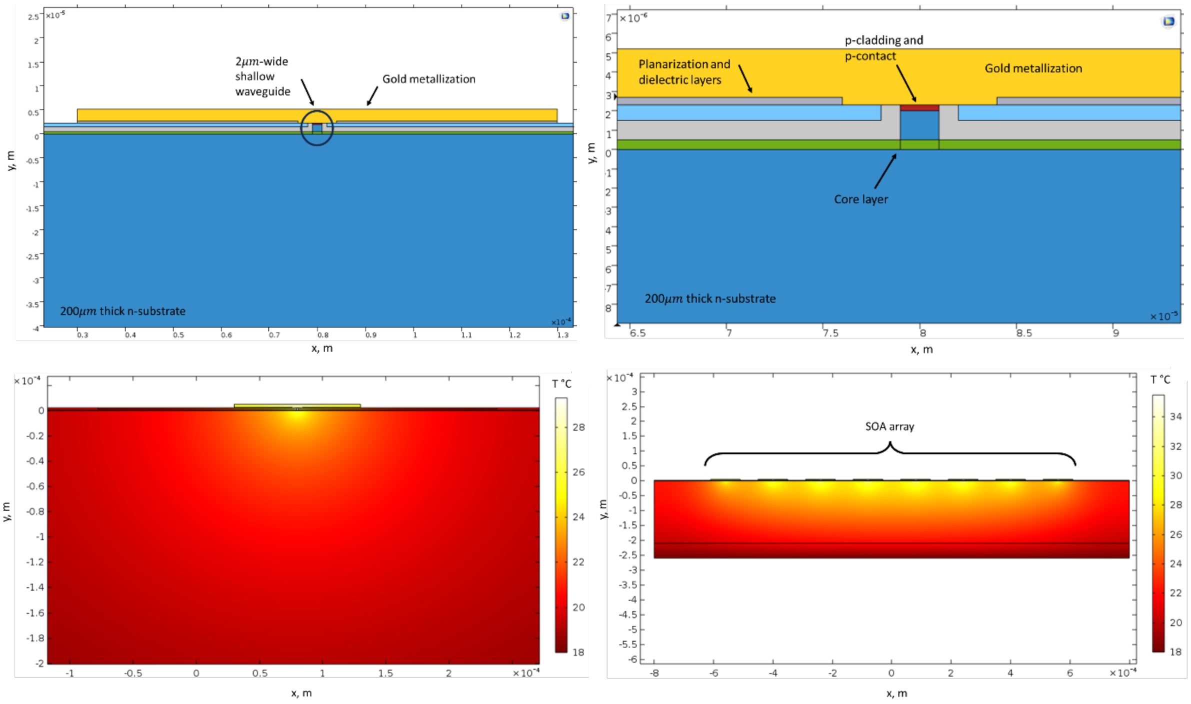

Supplementary Figure S4. (top) Transversal cross section showing the layer stack used to simulate the temperature profile in an SOA and zoom in of the waveguide cross section. Temperature distribution at the maximum current density of $10\ kA/cm^2$ for (bottom left) a single SOA, and (bottom right) the SOA array.

We simulated in COMSOL the average temperature in a single SOA waveguide (in its core and p-cladding) and compared it to the average temperature in an array of 8 SOAs with $160\ \mu m$ spacing (Supplementary Figure S4). The simulation assumes a $200\ \mu m$ thick substrate, a fixed substrate temperature of $18°C$, heat diffusion through the metal contact, and convective heat transfer in air at room temperature for the top PIC surface. The heat is generated through Joule heating at the p-contact and core layers of the waveguide supporting the SOA upon current injection. A contact resistance of $10\ \Omega$ was measured by the foundry, and we used it as a model parameter.

We estimate up to $6°C$ average temperature increase in the arrayed SOAs compared to a single SOA for the maximum considered current density of $10\ kA/cm^2$ (Supplementary Figure S5).

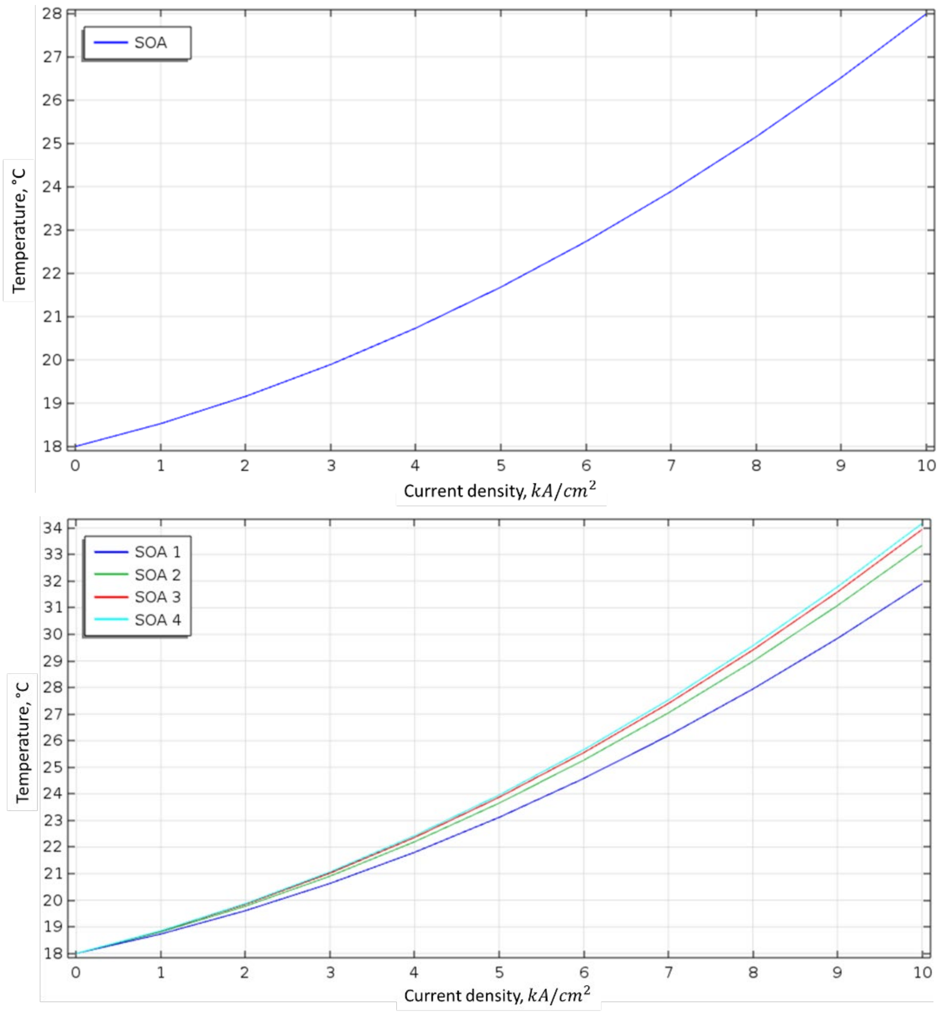

Supplementary Figure S5. Comparison of average temperature in a single SOA waveguide compared to an array of 8 SOAs (due to symmetry, only 4 SOAs are shown starting from the edge of the array to the center of it).

Furthermore, following the method described in [Supplementary Information 2], we measured the modal gain spectrum of an SOA at a fixed driving current density, $10.6\,kA/cm^2$, for different substrate temperatures (Supplementary Figure S6). The substrate temperature increase, and consequently, the increase of the active medium temperature, causes a decrease in the carrier density in the SOA multi-quantum wells contributing to a decrease of the modal gain peak and to a bandgap shift.

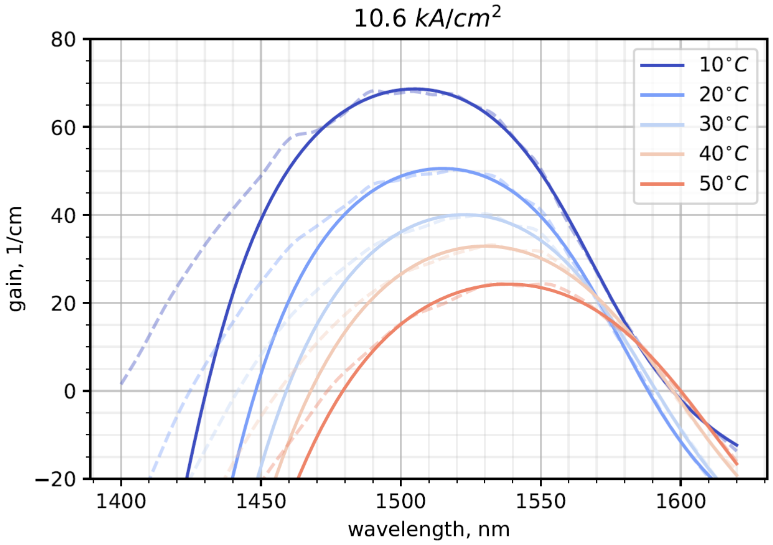

Supplementary Figure S6. Modal gain spectrum measured at different substrate temperatures (bottom, measured at $10.6\ kA/cm^2$ driving current). The dashed lines are measurement data; the solid lines are fitted curves. The measurement of the amplifier performance was done using a separate chip containing test structures for individual building block characterization.

For a $500\ \mu m$ long SOA, we estimate between 2 dB and 4 dB gain variations with an SOA temperature increase of $10°C$ (close to the simulated maximum temperature increase of $6°C$), at the peak wavelengths. Within the accuracy limits of the measurement and simulation techniques, these results are in accordance with the measured gain drop in the linear amplification regime shown in Figure 2 (c-f) of this publication. Moreover, the booster SOA's thermal influence was not considered within the scope of this publication to ease the complexity of simulations (justified because the booster is relatively far from the SOA array), but we expect a further drop in gain compared to the simulated one, due to the additional thermal crosstalk introduced by the booster.

4. Calibration and EOPM characterization

The calibration of the OPA phase distribution was carried out following the mREV method, as described in our previous work [iii]. The method we employed relies on fitting the sinusoidal far-field interference pattern between each OPA arm and the rest of the array to extract the phase-voltage relation of each EOPM. The results of this procedure are shown in Supplementary Figure S7.

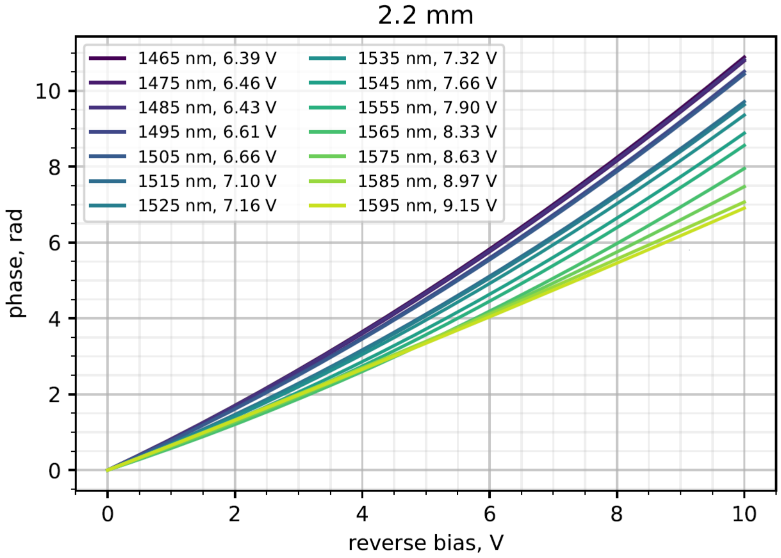

Supplementary Figure S7. Phase-voltage relation of the 8 EOPMs in the OPA for the wavelength range described in this publication. The relations are obtained through the calibration of the OPA. The plot's labels also show the voltages needed to reach 2π phase shift.

5. Near-field measurement of the OPA Gaussian distribution power ratio

Using the setup described in the Methods section of this work, we imaged the near-field OPA profile at different wavelengths of operation (Supplementary Figure S8). Moreover, we measured the power ratio between the inner and the two outer emitter elements and calculated the average between the two (i.e., average Gaussian distribution power ratio). The results of average power ratio as a function of wavelength are shown in Supplementary Figure S9.

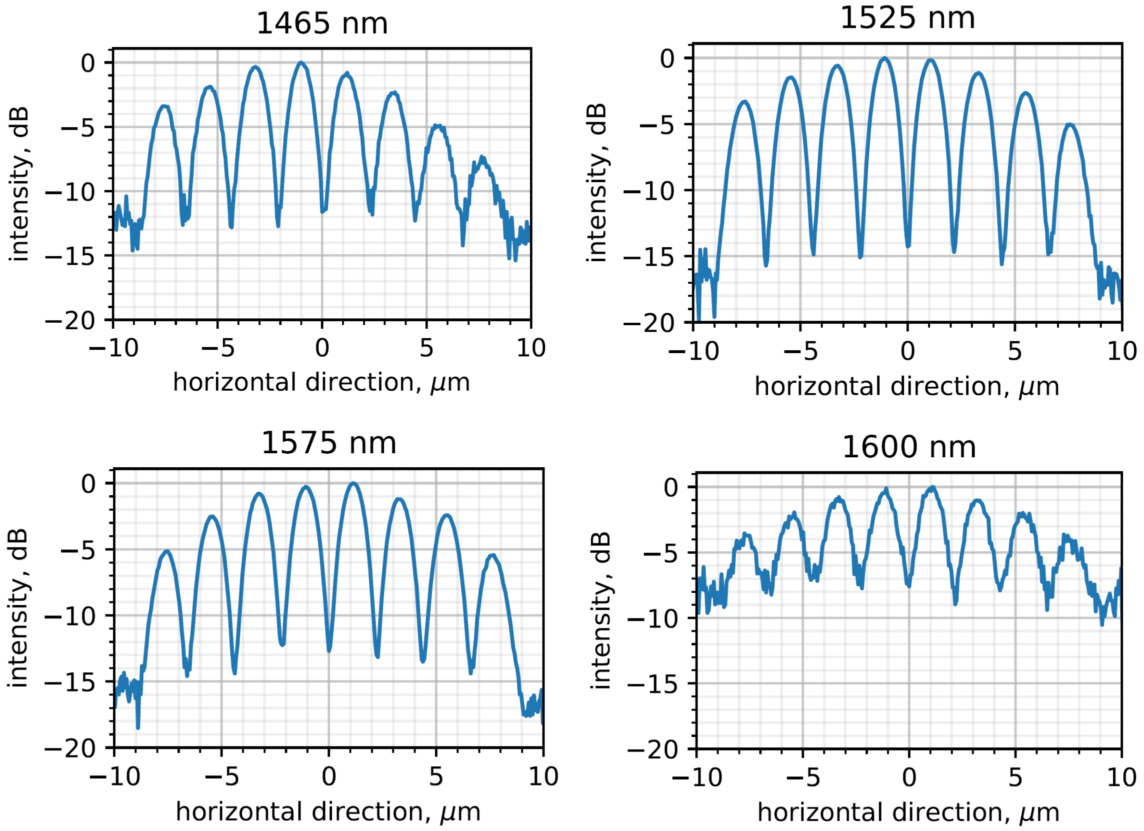

Supplementary Figure S8. 1D cross section of the measured near-field OPA profile. We measured the near-field profile at all wavelengths of interest for our study; here we show the measurement at 1465 nm, 1525 nm, 1575 nm, and 1600 nm.

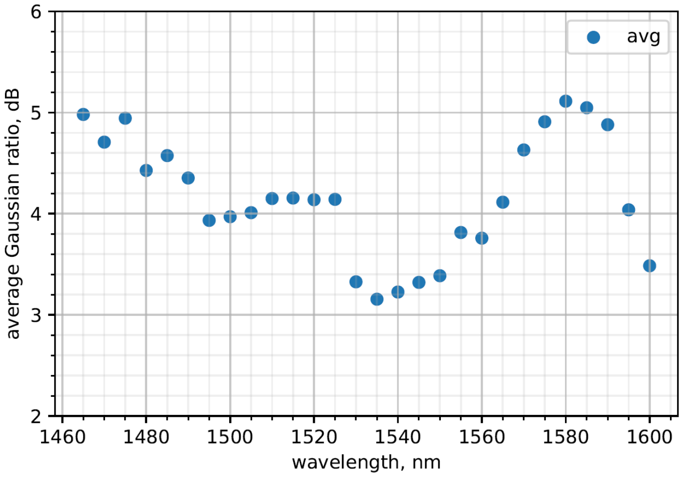

Supplementary Figure S9. Average Gaussian distribution power ratio as a function of wavelength of operation.

The Gaussian distribution power ratio trends with wavelength show a minimum close to the central wavelength (1525 nm), where the arrayed SOAs sub-linearity is the strongest, which leads to the leveling of the power levels in the OPA emitters.

At wavelengths that are longer and shorter than the central wavelength, there is less power coming from the booster due to the lower gain, so the arrayed SOAs move away from the sub-linear condition. The power profiles better match the star coupler's design value (6 dB).

For wavelengths longer than 1580 nm, the ASE becomes comparable to the peak power levels of the emitters. As a consequence, the measured Gaussian distribution power ratio drops to lower levels, in a similar manner to what is described for the SLSR of the far-field beam. For such wavelengths, we assume that the recorded Gaussian distribution power ratio values are limited by the measurement method, so we don't expect an influence on the far-field beam's FWHM, as it is shown in Figure 3 of the main article.

6. Simulations of far-field properties

Field of view

Through Lumerical FDTD simulations of the near-field and far-field, we estimated the OPA beam parameters. The field of view was calculated by simulating a far-field profile such that the phase difference in the emitters is π. The result is shown in Supplementary Figure S10 for 1550 nm wavelength, and for an emitter array having 6 dB Gaussian distribution power ratio. The field of view is measured as the difference between the two beams in the aliasing condition: $FoV = \pm 20.5°$.

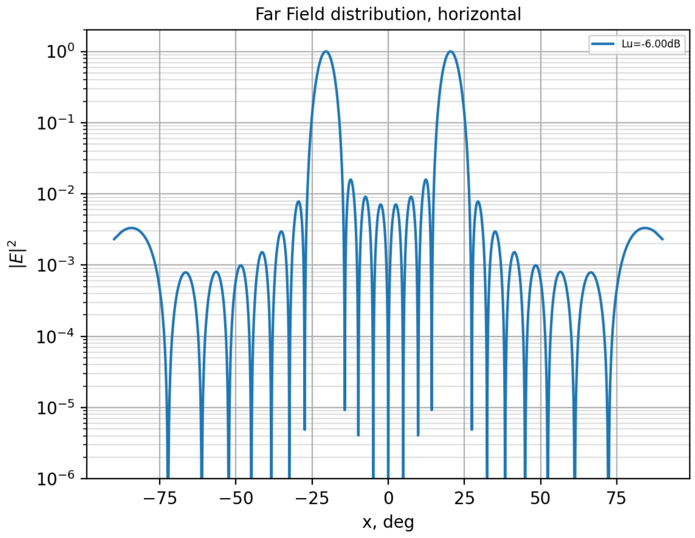

Supplementary Figure S10. Simulated far-field at the aliasing condition. The phase difference between consecutive arms is π, and the main beam is steered at the maximum angle in the field of view.

Far-field beam FWHM

The FWHM of the far-field beam is extracted for different wavelengths in the range of interest by simulating the far-field at a 0° steering angle. The results are shown in Supplementary Figure S11 for the Gaussian distribution power ratio value of the star coupler (6 dB), and the for a 3.3 dB power ratio, which was measured at the central wavelength. The simulation results show a linear dependence with the wavelength, as it is expected by approximating the main far-field lobe with a Gaussian beam.

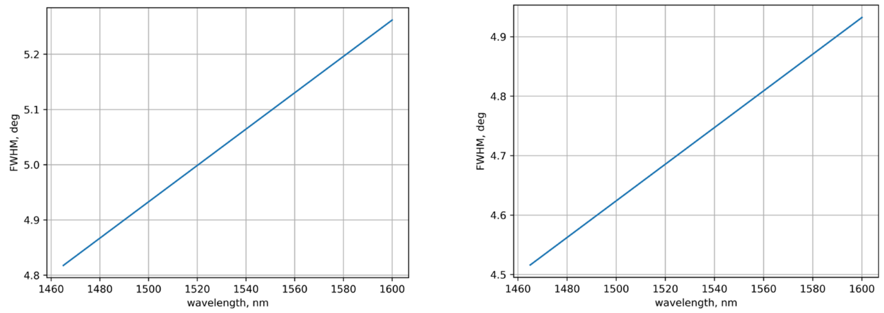

Supplementary Figure S11. Simulated FWHM of the far-field beam for a Gaussian distribution power ratio of (left) 6 dB and (right) 3.3 dB.

However, the OPA Gaussian distribution power ratio is not constant with the wavelength when the array of SOAs is operated close to sub-linearity. In this case, the sub-linearity will be maximum at the central wavelength, 1525 nm, where the modal gain of the SOAs is at its maximum (Supplementary Figure S2). By moving away from this condition, the booster SOA will output less power, which will bring the arrayed SOAs away from the sub-linear regime. In this case, the star coupler Gaussian distribution power ratio is better reproduced after amplification (Supplementary Figure S9).

We fitted the measured average Gaussian distribution power ratio with a quadratic polynomial (Supplementary Figure S12) and used such wavelength relation in the far-field simulations to extract the FWHM-wavelength relation that we measured and showed in Figure 3 of the main article. We excluded the wavelengths above 1580 nm from the fitting dataset because, as we explained in the previous section, the Gaussian distribution power ratio measurement was compromised by the higher levels of spontaneous emission radiation in free-space.

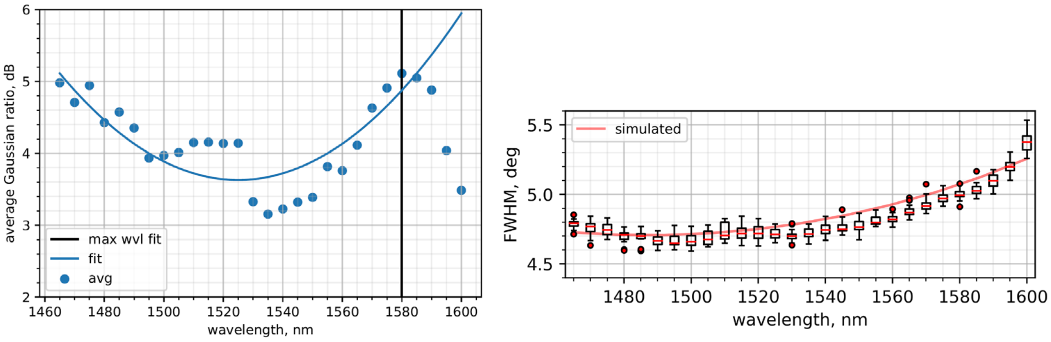

Supplementary Figure S12. Fitting of the measured Gaussian distribution power ratio data with a quadratic polynomial. The data for wavelengths above 1580 nm were excluded from the fit. The fit prediction for wavelengths between 1465 nm and 1600 nm was used in the far-field simulation to extract the FWHM shown in the right plot.

Far-field beam SLSR

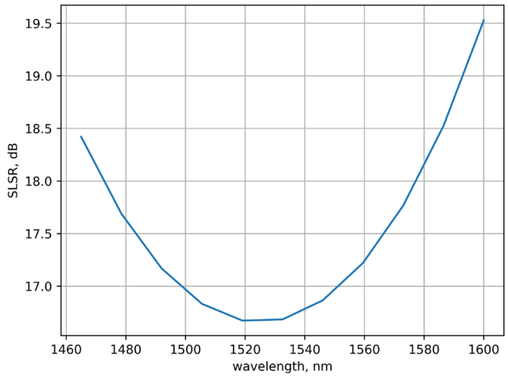

Supplementary Figure S13. Simulated SLSR as a function of wavelength. The Gaussian distribution power ratio extracted with the method described previously was used for each wavelength datapoint.

We calculated the SLSR of the far-field beam from the simulations described in the previous section. The wavelength relation of the Gaussian distribution power ratio extracted in the previous section was used. The SLSR values extracted from the simulation results are higher than the ones we measured, as discussed for Figure 4 in the main paper. In fact, there is a limitation in the measurement of the SLSR with the free-space setup and IR camera we employed due to the spontaneous emission levels and background radiation collected by the camera.

Data availability

The datasets generated during and/or analysed during the current study are available from the corresponding author on reasonable request.

---

[i] Hofstetter, D. & Thornton, R. L. Theory of loss measurements of Fabry–Perot resonators by Fourier analysis of the transmission spectra. Optics Letters vol. 22 1831 (1997).
[ii] Pustakhod, D., Williams, K. & Leijtens, X. Fast and robust method for measuring semiconductor optical amplifier gain. IEEE J. Sel. Top. Quantum Electron. 24, 1–9 (2018).
[iii] Gagino, M., van Rijn, M. B. J., Bente, E. A. J. M., Smit, M. K. & Dolores-Calzadilla, V. Broadband operation of an InP optical phased array. IEEE Photonics Technol. Lett. 34, 541–544 (2022).
